# Influence of Mothers

**Published:** 1863-02

**Authors:** 


### INFLUENCE OF MOTHERS.

John Randolph never ceased, till his dying day, to remember with unutterable affection the pious care of his mother, in teaching him to kneel at her side, and, with his little hands pressed together, and raised upwards, to repeat, in slow and measured accents, the pattern prayer.

"My mother," said Mr. Benton, not long before he died, "asked me not to drink liquor, and I never did. She desired me at another time to avoid gaming, and I never knew a card. She hoped I would not use tobacco, and it never passed my lips."

Not long ago, the Rev. Dr. Mills, in one of his powerful appeals to mothers to consecrate their children to the ministry of the Gospel, said: "A youth, after great deliberation, and with the knowledge that his mother desired him to be a clergyman, decided at last to become a lawyer; and, soon after, his mother inquired of him, in a tone of deep and tender interest, 'My son, what have you decided to do?' 'To study law, mother.' She only replied, 'I had hoped otherwise;' and her convulsive sobbing told the depth of her disappointment. 'Do you think,' said he, 'I could go into the law over my mother's tears?' He reconsidered the case, and has long been an able and efficient clergyman.

All that Leigh Richmond was, he attributed to the simplicity and propriety with which his mother endeavored to win his attention, and store his memory with religious truths, when yet almost an infant.

Oh! if Christian mothers would but wake up to the use of their powers and their influences, a Samuel might arise out of every family, and Leigh Richmonds be numbered by thousands!

